# A retrospective study regarding the influence of COVID-19 disease on asthma

**DOI:** 10.1186/s12890-023-02309-7

**Published:** 2023-01-17

**Authors:** Ioana Adriana Muntean, Polliana Mihaela Leru, Irena Pintea, Ioana Corina Bocsan, Carmen Teodora Dobrican, Diana Deleanu

**Affiliations:** 1 Allergology Department, Department of Allergology and Immunology and “Octavian Fodor” Institute of Gastroenterology and Hepatology, “Iuliu Hatieganu” University of Medicine and Pharmacy, 400347 Cluj-Napoca, Romania; 2 Clinical Department 5, “Carol Davila” University of Medicine and Pharmacy, Bulevardul Eroii Sanitari 8, 050474 Bucharest, Romania; 3 Department of Pharmacology, Toxicology and Clinical Pharmacology, “Iuliu Hatieganu” University of Medicine and Pharmacy, 400347 Cluj-Napoca, Romania

**Keywords:** Asthma, COVID-19 pandemic, Chronic diseases, Long-term care, SARS-CoV-2 infection

## Abstract

**Background:**

During the Covid-19 pandemic patients suffering from asthma raised many concerns regarding the outcome ofthe impact of COVID-19 disease on their preexisting condition. The 2021 GINA report indicates that people with asthma do not appear to be at increased risk of a severe form of COVID-19.

**Method:**

This study is a retrospective study of patients (n = 163) median age = 27.8 years, M:F = 1:1.26, with asthma evaluated using ACT (asthma control test) and VAS (visual analog scale) before and after COVID-19 disease. An ACT score over 20 points placed patients in the controlled asthma group.

**Results:**

The overall evaluation for COVID-19 in our asthma patients revealed that 22.7% of the studied group had the COVID-19 disease (21.5% in the controlled asthma group and 24.5% in uncontrolled asthma group). Asthma disease history was longer in the uncontroled asthma group (128 ± 96.8 months vs. 296 ± 59.7 months, *p* = 0.05). Asthma treatment was conducted according to the GINA guideline, and 18.4% (30 pts) of the patients were on allergen immunotherapy treatment. Significantly more uncontrolled patients were significantly more in Step 1 and 5 of treatment (*p* = 0.05 and *p* = 0.03). During the COVID-19 pandemic, patients in the GINA step 5 of treatment experienced a worsening of asthma, often twice as severe as compared to patients with asthma in GINA step 1–4. In these patients, even mild COVID-19 disease led to worsened asthma symptoms, while severe COVID-19 led to a severe asthma impairment measured by ACT score (*p* = 0.03) and VAS scale (*p* = 0.02), with increased oral corticosteroids consumption.

**Conclusion:**

Maintaining optimal asthma control should be able to reduce risk of severe outcomes after COVID-19 disease. Communication via phone with the specialist involved in their asthma care was very comforting for patients, thus confirming the necessity to include phone calls, smart phone’s application or online evaluations and counseling in long-term care of chronic diseases.

## Background

Asthma is a heterogenous disease, usually characterized by chronic inflammation of the airways. It is defined by the history of respiratory symptoms such as wheeze, shortness of breath, chest tightness, cough that may vary over time and in intensity, together with the variable airflow limitation [1]. During the Covid-19 pandemic, patients suffering from asthma raised concernes and raised many questions regarding their increased risk in case of SARS-CoV2 infection [2]. The 2021 GINA report and the recent metanalysis indicate that people with asthma do not appear to be at increased risk of acquiring the SARS-CoV2 infection or having a severe form of disease or an increased death rate due to the COVID-19 [1, 2]. The risk of death is related to the recent course of oral corticosteroids administered for uncontrolled asthma [3].

The COVID-19 disease is an acute respiratory syndrome that emerged in the city of Wuhan and rapidly spread throughout the world causing a global pandemic. The Center for Disease Control and Prevention as well as the American Academy of Allergy, Asthma & Immunology consider asthma a risk factor for severe COVID-19 [4]. Coronaviruses are among the top 5 viruses isolated during acute asthma exacerbations, with a higher prevalence in adults [5]. The COVID-19 disease presents respiratory symptoms, from mild to severe, and a significant percentage of patients develop acute respiratory distress syndrome (ARDS) with the Delta variant of the virus and less with Omicron. Severe symptoms are associated with a true cytokine storm, in particular elevation of IL-6, having death as one of the outcomes [6]. Old age and underlying morbidities, such as cardio-vascular diseases, in particular hypertension and metabolic disorders (obesity and diabetes), have been identified as significant risk factors for COVID-19 morbidity and mortality [7, 8]. Unlike asthma, which is not included in the common comorbidities for COVID-19, COPD is considered a risk factor for worse outcomes when infected with SARS-CoV-2. Therefore, COPD is one of the most common comorbidities worldwide, besides other important comorbidities such as chronic kidney diseases (CKD) under conservative treatment or renal replacement therapy (RRT) [9, 10]. The real impact of SARS-CoV-2 on asthma control is still unclear and may be variable in different countries and subgroups of patients.

The current understanding of the interactions between SARS-CoV-2 and asthma is still in the early stages, while observational and experimental data are still awaited to elucidate the relationship between COVID-19 and asthma [11]. Considering the relatively high prevalence of asthma, it is reasonable to hypothesize that asthmatic individuals are relatively resistant to COVID-19 because of the disease characteristics and/or the conventional treatment for asthma. Those patients also protected themselves more using social distancing and facial mask, thus experienced less asthma exacerbations due to distinct viruses [1–4]. The aim of this paper is to assess how asthma patients managed their disease during the pandemic, being monitored by their specialist through based on phone calls or emails and to evaluate the impact of the SARS-CoV-2 virus infection on asthma outcome.

## Methods

### Study design

This study is a retrospective study of patients (n = 163) with asthma diagnosis treated in a public institution: Allergy Department of the Professor Doctor Octavian Fodor Regional Institute of Gastroenterology and Hepatology, (Cluj-Napoca, Romania), between February 2017 and January 2022. The study protocol was approved by the “Octavian Fodor” Institute of Gastroenterology and Hepatology Ethics Committee (Approval no. 364/11.01.2021), and all the patients signed the informed consent before any assessments were done. Inclusion criteria was the diagnosis of asthma according to the GINA guideline, for at least 1 year. The exclusion criteria were as follows: COPD, systemic autoimmune diseases, chronic infections (e.g., viral B hepatitis, viral C hepatitis which continue to be widespread in Romania) [12].

The total number of patients in our Allergy clinic exceeds 2500 patients/year (outpatient and hospitalized) in the Allergy Department. In 2020 due to the pandemic restrictions there were only 1700 patients. The prevalence of asthma patients among our patients, during the 5 years of evaluation was 1.4%. In Romania the prevalence of asthma according to the ISAAC study was over 15% in school age children, with no data available in adults [13].

### Patients and clinical evaluation

Asthma was diagnosed according to the GINA guideline based on, history of typical symptoms: wheeze, shortness of breath, chest tightness, cough, and spirometry with a positive bronchodilator test of 12% and 200 ml increased of FEV1. All the asthma patients over 5 years old were included in this retrospective study. The studied group included 1% of allergic asthmatic children, but the main target patients were adults and adolescents over 12. Patients were from urban areas, but the social-economic status or the environmental exposure to allergen or tobacco smoke was not evaluated in the present study.

The asthma diagnosis was made at least 1 year before the date of the inclusion in the present study. The diagnosis included a previous spirometry with positive bronchodilator test when FEV1 was under 85% from the predicted value. Since spirometry was considered a nebulization method which may increase viral particle spreading, in asthmatic patients who were monitored during COVID-19 pandemic, performing spirometry was not in accordance with international recommendations. Age, sex, and residence (rural/urban), sensitization for inhaled allergen and asthma related symptoms were recorded. Patients were assessed for asthma control monthly using Visual Analog Scale (VAS) for QoL (quality of life) assessment, which was a simple and easy to understand method. Interviews with the patients were by phone or online as well as face to face duringclinical examination in severe symptomatic patients. In all patients with asthma the stepwise treatment used was according to the GINA guideline [14].

The Asthma Control Test (ACT) is a friendly and accurate asthma control evaluation tool for adults and children. Based on their ACT results patients were included in 2 groups: controlled (ACT ≥ 20 points) and uncontrolled (ACT < 20 points) [15]. Also, VAS scale was used to assess the QoL, in Allergic rhinitis, though it can be used in various chronic diseases [16].

### Patients evaluation during the pandemic

All the patients were called or e-mailed, and asked tocomplete VAS and ACT forms, monthly, at home as showed in Table 1. They later sent the results by text message or e-mail. Some of the patients required a face-to-face consultation following the COVID-19 disease, due to asthma severity, to step-up asthma treatment according to GINA recommendations. The asthma outcomes used in the present study were based on subjective methods like the ACT questionnaire and the VAS scale. ’Improvement’ was defined by a 3-points ACT increase, ‘unchanged’ was defined as a ± 2-point ACTchange, and ’worsening’ or ‘exacerbation’ corresponded to a 3 points ACT decrease or in case when hospitalization for respiratory symptoms was required.

**Table 1:** Questions asked during online or phone consultation

Question	Response
*ACT *	1–5 points
1. In the last 4 weeks, how much of your time did your asthma keep you from getting as much done at work, at school or at work?	
2. In the last 4 weeks, how often have you had shortness of breath?	
3. In the last 4 weeks, how often did your asthma symptoms (wheezing, coughing, shortness of breath, chest tightness) wake you up at night or earlier than usual in the morning?	
4. In the last 4 weeks, how often have you used your rescue inhaler or nebulizer medication (salbutamol/formoterol + ICS)?	
5.How did you rate your asthma control during the past 4 weeks?	
*VAS *	0–10 points
How much has your asthma had bothered you (0- no bothersome to 10- extremely bothersome)	
*Presence of COVID-19 disease in the last month *	Yes/No

### Skin prick tests (SPT)

The diagnosis of allergy was established through a skin prick test, according to international guidelines [17]. The allergen panel included international recommendation and particularities of exposure to the following allergens in Romania: Dermatophagoides pteronyssinus, Dermatophagoides farinae, grass pollens, cereals pollen, birch pollen, hazel pollen, cat and dog dander, Alternaria Alternata, Artemisia vulgaris and Ambrosia elatior. SPT was performed at the beginning of the study using standardized allergen extracts (Hal Allergy, Netherlands).

### The COVID-19 disease evaluation

COVID-19 disease severity was defined by using the scale provided by the World Health Organization (SpO2 < 94% on room air at sea level, PaO2/FiO2 < 300 mm Hg, a respiratory rate > 30 breaths/min, or lung infiltrates > 50%) [18]. In our clinic we just evaluated the asthma after the infection, the Covid-19 severity diagnosis was taken from medical fies. All the patients with asthma who had Covid-19 disease symptoms were diagnosed using PCR for COVID-19, laboratory tests and CT-scans in a COVID-19 Department at the Infectious Disease Hospital from Cluj-Napoca, not in our Department of Allergology. We analyzed the data from patient’s files only retrospectively, calling or emailing the patients to obtain data and the medical documents they received after the evaluation in the COVID-19 Department at the Infectious Disease Hospital. Laboratory tests (complete blood count, CRP, e.g.) were evaluated in our department after 14 days of quarantine, though it was not possible to get tests for all the patients because some with mild forms did not go to hospital, as only GPs evaluated and diagnosed them. All the patients were called and were advised to inform our office if they suspected SARS-CoV-2 infection.

### Biological evaluation

The blood tests results obtained à jeun in all patients were: complete blood count including eosinophils by using the SYSMEX-XN-1000 analyzer and CRP, LDH, Troponin tests by using the COBAS PRO C 503/E 801analyzer. The lab test values were obtained from patient’s files between day 10 and day 15 after the positive SARS-CoV-2 RT-PCR was confirmed or by the direct evaluation of 30 patients with mild forms of COVID-19 disease on the 15th day.

### Statistical analysis

The statistical analysis was performed using the SPSS version 21 (Chicago, IL, USA) and Microsoft Excel. Data was labeled as nominal, expressed as percentages, and continuous variables. The differences were assessed within groups by the Wilcoxon Signed Rank test and between groups by the Student t test. The Spearman’ coefficient of correlation was calculated to highlight differences between continuous variables. The level of statistical significance was set at *p* < 0.05.

## Results

**Fig. 1:**
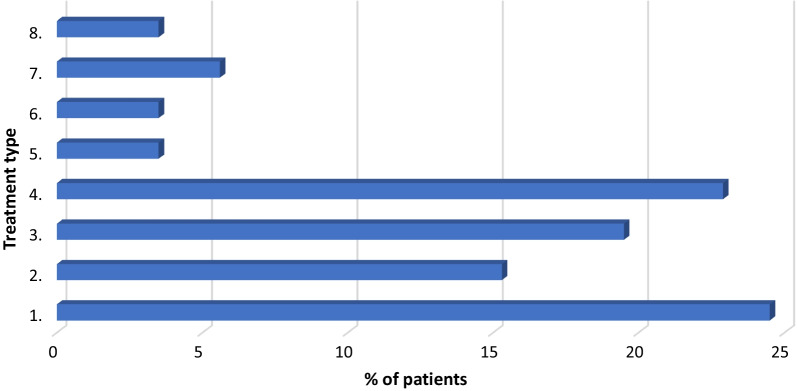
Pharmacological treatment use for asthmatic patients according to GINA guideline. x-axis (%): number of patients on mentioned type of medication; y-axis (type of medication): (1) as needed ICS and LABA/SABA, (2) ICS low-dose or montelukast, (3) ICS low-dose and LABA, (4) ICS medium-dose and LABA, (5) ICS high-dose and LABA, (6) ICS high-dose and LABA and LAMA, (7) ICS high-dose and omalizumab, (8) ICS high-dose and benralizumab

Patients’ demographic data are presented in Table 2. The treatment data of the studied group of asthma patients during SARS-CoV-2 pandemic (February 2020–April 2021) are presented in Fig. 1. All the asthmatic patients aged over 5 years were included in this study. The Median age was 27.8 (5–85) years, and the sex ratio M:F was 1:1.26. The 73 patients who had ACT score equal or greater than 20 were included in the controlled asthma group and 90 patients (55.2%) were included in uncontrolled asthma group. All patients had undergone SPT for inhaled allergens during their initial evaluation, according to the international guidelines. Of the total number of patients, there were only 9.8% non-atopic, lower than the reported 40% non-atopic ones in the Step-5 GINA guideline.

Asthma treatment was administered according to GINA guideline, and 18.4% (30 pts) of the patients were on allergen immunotherapy treatment (AIT) of which 80% for house dust mites and 20% for other allergens (cat, pollens). In Romania, AIT is not reimbursed by the National Insurance System, so just few of the allergic patients afforded to pay for the treatment, due to socio-economic status. No impairment in asthma symptoms was registered in patients with AIT who had COVID-19 disease.

**Table 2:** Demographic data of the patients

Parameter	Controlled asthma (n = 73)	Uncontrolled asthma (n = 90)	*p*
Age	21.05 ± 10.2	29.59 ± 19.7	0.6
Sex			
Male	61.6% (45)	30% (27)	0.01
Female	38.4% (28)	70% (63)	0.01
Living area			
Urban	78.2% (62)	68.4% (69)	0.7
Rural	21.8% (11)	31.6% (21)	0.8
Asthma duration (months)	128 ± 96.8	296 ± 59.7	0.05
Rhinitis present	80.8% (59)	77.7% (70)	0.9
Asthma scores			
ACT	22 ± 2	13 ± 5	0.01
VAS	8 ± 2	3 ± 4	0.01
Asthma GINA steps treatment			
Step 1	9.6% (7)	36.6% (33)	0.05
Step 2	19.2% (14)	12.2% (11)	0.8
Step 3	34.2% (25)	6.8% (6)	0.04
Step 4	27.4% (20)	21.1% (19)	0.9
Step 5	5.6% (4)	23.3% (21)	0.03
COVID 19 infection			
No present	79.5% (58)	75.5% (68)	0.9
Present	21.5% (15)	24.5% (22)	0.6

Statistical significance is at *p* ≤ 0.05

### The COVID-19 disease in Asthma Patients

The overall evaluation for COVID-19 disease in our asthma patients showed that only 22.7% of the studied group were infected (21.5% in the controlled asthma group and 24.5% in uncontrolled asthma group). Of the COVID-19 cases, 80% were mild. No asthma exacerbation was observed in allergen immunotherapy group was observed.

The inflammatory markers in COVID-19 of asthma patients are described in Table 3, showing significant statistical differences between mild and moderate/severe forms of disease.

**Table 3:** Covid-19 disease in asthma patients’ group

Parameter	Mild COVID-19 infection n = 30	Moderate/severe COVID-19 infection n = 7	*p*
Leucocytes 10^3^/µL	10.54 (± 9.4)	4.59 (± 2.1)	0.03
CRP (mg/dl)	4.2 (± 5.1)	68.7 (± 42.6)	0.01
Troponin (µg/L)	0.1 (± 0.2)	2.3 (± 1.8)	0.04
LDH (U/l)	538 (± 175.6)	757 (± 195.8)	0.04
Eosinophils 10^3^/µL	0.35 (± 0.2)	0.26 (± 0.21)	0.06
ACT	19 (± 5.2)	12 (± 3.8)	0.03
VAS	9 (± 0.8)	7 (± 1.4)	0.02

Data are expressed as mean ± SD. Statistical significance is at *p* ≤ 0.05

Three patients in omalizumab group and one patient in benralizumab group presented SARS CoV-2 infection, with a mild to moderate form of disease and one hospital admission (non-vaccinated patient) in omalizumab group. The non-vaccinated 80 years old patient died 6 months later due to stroke, and this was the only death reported in our asthmatic patients group. All the patients experienced a worsening of asthma symptoms and received a 2–3-week course of oral corticosteroids or a step up if they were in step 1–4.

We use phone consultation to evaluate the patients and all our patients had an emergency kit for asthma exacerbation at home containing oral corticosteroids, and, in hospitalized patients a phone contact with the doctor involved in the case was maintained.

Patients in the GINA step 5 of treatment experienced a worsening of asthma, often twice as severe as compared to patients with asthma in GINA step 1–4. In patients with mild COVID-19 the worsening of asthma was treated with step-up treatment according to GINA. Moderate/severe COVID-19 cases were treated according to WHO recommendation in infectious clinic diseases, the treatment including oral corticosteroids, antibiotics, and oxygen. Asthma course developments during COVID-19 disease is described in Tables 4 and 5. Due to similar symptoms of asthma exacerbation and COVID-19, such as dyspnea, cough and chest tightness, worsening of asthma symptoms was hard to evaluate. No asthma related deaths were reported in this group. Patients in GINA step 5 of treatment (high dose ICS with/without biologicals) were 80% vaccinated, and 20% non-vaccinated. In the non-vaccinated group there was a non-asthma related death 6 months after COVID-19 infection and none in vaccinated group.

**Table 4:** Asthma course influenced by COVID-19 disease

Asthma course	Mild COVID-19 disease (n = 30)	moderate/severe COVID-19 disease (n = 7)
Improvement	1	0
No change	13	0
Uncontrolled/Exacerbation	16	7

**Table 5:** Asthma in step 5 GINA treatment course influenced by COVID-19 disease

Asthma in step 5 GINA course	mild COVID-19 disease (n = 19)	moderate/severe COVID-19 disease (n = 5)
Improvement	0	0
No change	6	0
Uncontrolled/Exacerbation	13	5

## Discussion

Asthma is a heterogenous disease with an important social impact and increased costs. Many factors, mainly viral infections, can lead to asthma exacerbations. [1, 19]. The main goals of asthma management are to optimize control of asthma symptoms and to reduce the risk of asthma exacerbation and hospitalization, while minimizing medication adverse effects, especially referred to oral corticosteroids [20]. Asthma is not included in the risk factors for severe COVID-19, but the impact of SARS-CoV-2 virus and its variants on asthma is still being studied, some of the available research showing that asthma may complicate the COVID-19 and conversely [21].

Coronaviruses respiratory infection may lead to asthma exacerbation. It is still unclear how and if SARS-CoV-2 influences the outcome of asthma patients. [10, 20, 21]. Currently, the patients with underlying moderate to severe asthma are a risk group for severe COVID-19 and/or asthma exacerbation [22] mainly evaluated in Delta variant of the virus and less in Omicron. For patients with asthma there was an overlap with the symptoms of COVID-19, including cough, shortness of breath and chest tightness, consequently it was difficult to distinguish these from those of severe asthma exacerbation [23].

The role of mast cells (MCs) in coronavirus-induced disease have been discussed since the beginning of the Toll-like receptor 3 detection of viral double-stranded ribonucleic acid (RNA), viral sphingosine-1-phosphate (S1P) binding to S1P receptors, and retinoic acid-induced gene I (RIG-I) recognition of uncapped viral RNA). Mast cells express angiotensin converting enzyme 2 receptor (ACE2), now known as the principal receptor for SARS-CoV-2, thus defining a route by which mast cells could also become one of the hosts for this virus, and exacerbate mast-cell related diseases [21–23]. Some researchers claim that allergic diseases could be protective in terms of infection severity from COVID-19, which could be explained by the evidence that ACE-2 receptor is down-regulated in allergic patients, including allergic asthmatics [24, 25]. On the other hand, some epidemiological studies indicate that asthma and allergies are comorbidities for severe COVID-19 forms [10]. Another question raised in asthmatic patients was the possible protective role of eosinophils in terms of SARS-COV-2 infection. Patients with asthma may be at a reduced risk of poor outcomes from COVID-19 infection. Eosinophilia, both in those with and without asthma, may be associated with reduced mortality risk, as Ho et al. showed in their study. But in severe asthmatics, defined by oral corticosteroid use in the previous year, it has been observed a slightly increased hazard ratio of mortality despite eosinophilia. [26] In our asthma patients’ group there were 6 patients treated with add-on benralizumab, which is an eosinophil depleting biologic treatment. Only one patient in this group had a mild form of COVID-19 disease, with no need of oral corticosteroids course, similar to another case described in the literature [27].

From our group of asthma patients only 22.4% presented COVID-19 disease which is similar with other studies [26]. In the group with uncontrolled asthma, 24.5% of the patients had COVID-19, and there were no statistical differences in terms of getting the infection between controlled and uncontrolled asthmatics. However, it is still important to maintain good asthma control, as poorly controlled asthma may lead to a more complicated COVID-19 course, and some studies have found a higher rate of intubation and prolonged mechanical ventilation in adults with asthma [21]. All the asthma drugs should be available, including inhaled glucocorticoids, long-acting bronchodilators, oral glucocorticoids, and biological agents approved for asthma, and should be continued to be administered during the COVID-19 pandemic [3, 20–23]. Maintaining good asthma control helps minimize the risk of asthma exacerbation as other studies have shown [23, 28]. The group of uncontrolled asthma patients in our study representing 55.2%, was probably due to persistent inflammation in the lower airways even before symptoms appeared [29]. Preexisting airway inflammation may lead to exacerbation during a viral respiratory infection or in presence of other triggers, as other studies showed inflammatory diseases exacerbations during and after COVID-19 [1, 30]. This could be due to failure to acknowledge the asthma symptoms or secondary to minimizing them. Another problem was the high cost of medication without reimbursement, due to logistical problems in long-term care of chronic diseases during the pandemic.

Despite respecting the recommendations according to GINA guidelines, only 44.8% patients from our group were controlled, with ACT score over 20 points. There were more controlled asthma patients in GINA Step 2 and Step 4 and less in GINA Step 1, 3 and 5 there were less controlled. We may hypothesize that in Step 1 of treatment with only as needed medication (low dose ICS/formoterol or salbutamol followed by low ICS dose) patients tend to reduce medication use due to underestimation of their symptoms. Poor asthma control is a risk factor for greater severity of viral-induced exacerbation [1]. Asthma control is difficult to achieve in Step 5 which includes a high ICS dose and different add-on therapies, including biologicals. In their study Racine et al. showed that uncontrolled asthma, smoking, and psychological distress are risk factors for asthma exacerbation [31].

The influence of COVID-19 on asthma in our group showed a worsening of asthma course in 62.1% of infected asthmatics, because even mild COVID-19 could lead to asthma exacerbation, needing a step-up treatment according to GINA guidelines. In our group of patients with severe COVID-19, we observed that there was a statistically significant increase of inflammatory markers correlated with decrease of control evaluated by ACT score and decrease in QoL evaluated by VAS scale. Therefore, maintaining optimal asthma control should be able to reduce the risk of severe outcomes in COVID-19, like Jackson et al. showed in their study since 2015 [32]. Even though over half of the patients included in our study had uncontrolled asthma, no COVID-19 related deaths were reported.

The clinical evaluation and spirometry were difficult to maintain in our country during the pandemic, only patients that underwent a biological treatment or subcutaneous AIT were evaluated monthly (omalizumab) or at 2 months (benralizumab after the first 3 months). The possibility of maintaining phone contact with the specialist involved in their asthma care was very comforting for patients with asthma. This may lead to the necessity to include phone-calls, smart phone’s application or online evaluations and counseling in asthma patient’s care [33]. In our study all asthma patients had the possibility to reach their physician by phone or e-mail. The patients were educated to recognize and to self-manage their asthma exacerbation using ICS/formoterol treatments, salbutamol and ICS or OCS as stated in the GINA guideline recommendations are [1].

Another problem raised during pandemic was the influence of inhaled cortico-steroids on COVID-19 disease. There is no solid evidence that inhaled glucocorticoids, or the biological agents used for asthma, which do not have a systemic immunosuppressive effect, have an adverse effect on the course of COVID-19 [33–35]. In our study, we noticed that patients in GINA Step 1 with as needed treatment experienced more often asthma exacerbations than patients with daily intake of ICS, which shows a protective effect for asthma exacerbation. The results from two studies indicate that individuals with nonallergic asthma have a higher risk for severe outcome of COVID-19 than those with allergic asthma [21, 25]. In our study we mainly included patients with allergic asthma, thus probably influencing the good outcome (no death reported after COVID-19 disease) could be influenced by atopic status or genetic polymorphisms, which are not studied in our population.

In the allergen immunotherapy (AIT) group, no asthma exacerbation was noted, since AIT was not discontinued according to international recommendation [36]. Therefore, asthma was controlled in this patients’ group, raising no concerns, as was the case in similar groups of asthma phenotypes [37]. Patients with severe asthma, uncontrolled in step 5 GINA treatment are at higher risk for severe outcome, those patients experienced a worsening of asthma symptoms twice as often compared to asthma patients in GINA step 1–4. From our infected patients’ group, 64.8% were in Step 5 GINA treatment, which support other study findings proving that severe asthma is influenced by COVID-19 [38]. No death was reported in the severe asthma patients’ group even when they had a severe impairment of the lung function. In the omalizumab group which also included also an un-vaccinated old patient, there was a protection despite the severe COVID-19, as other studies showed regarding the use of omalizumab in allergic diseases showed [39, 40]. Biological treatment may act protectively via several pathways. Omalizumab prevents IgE from binding to its receptor on plasmacytoid dendritic cells, leading to lower IFN-1 production by cross-linking of IgE [41]. In conjunction with asthma and COVID-19 severity, it was suggested in other studies that those with more severe asthma who require high dose of inhaled corticosteroids (Step 5 GINA) to maintain asthma control may be at risk for worse prognosis from COVID-19 [1, 22, 37].

As a first limitation of this study we should mention that the laboratory tests, SpO2 values and imaging of asthma patients before COVID-19 were not available, so it was not possible to evaluate those parameters before and after COVID-19. The second limitation was the difficulty to discriminate respiratory symptoms such as dyspnea, cough, and chest tightness due to worsening of asthma from those caused by COVID-19. Another limitation of our study is the subjectivity of the outcome measures, given that during the pandemic spirometry was not recommended, being considered a nebulization method which might increase the viral particle spreading. Another limitation is that we had a retrospective study, which is also less powerful than other types of studies, such as prospective studies. Strength of our study consists in a large group of asthma patients diagnosed and monitored before the COVID-19 disease who were evaluated with asthma control test questions during the pandemic. Although, the COVID-19 immunization through vaccination was not our study’s topic, patients with asthma are recommended to receive the COVID-19 vaccination [42]. In our group of asthmatics only one death was reported in a non-vaccinated patient, that was a non-asthma related death 6 months after COVID-19 infection and none in the vaccinated group, but no conclusion can be drawn due to the small sample size. According to the current information we cannot estimate if there was a selection bias for survival among this asthma patient population especially because the study is a retrospective one.

## Conclusion

We conclude in our study that the influence of COVID-19 on asthma may lead to the worsening of symptoms mainly in moderately severe COVID-19 cases and in uncontrolled asthma. Therefore, in asthmatic patients, besides every effort that should be made to avoid exposure to the SARS-CoV-2 virus, all regular medications necessary to maintain asthma control should be available and be used. Maintaining optimal asthma control should be able to reduce risk of severe outcomes in COVID-19 disease, therefore asthma even uncontrolled is not a risk factor for COVID-19 related fatalities in allergic asthma patients. The possibility of phone contact with the specialist involved in their asthma care was very comforting for patients, thus confirming the necessity to include phone calls, smart phone’s applications or online evaluations and counseling in long-term care of chronic diseases.

## Data Availability

Data are available at Allergology Department, Octavian Fodor” Institute of Gastroenterology and Hepatology, Cluj-Napoca.

## Declarations

### Declarations

All the patients or parents for patients under 16 years old signed the informed consent before any medical data were collected for the study according to the Declaration of Helsinki.
